# Research on Monetary Policy Implementation and Industrial Structure Transformation Under COVID-19—Evidence From Eight Economic Zones in Mainland China

**DOI:** 10.3389/fpubh.2022.865699

**Published:** 2022-05-20

**Authors:** Baicheng Zhou, Shu Wang, Henan Gao, Han Wang

**Affiliations:** ^1^China Center for Public Sector Economy Research, Jilin University, Changchun, China; ^2^School of Economics, Jilin University, Changchun, China

**Keywords:** COVID-19, monetary policy, industrial, economic zones, China

## Abstract

The outbreak of COVID-19 has brought a serious impact on the economies of various countries, monetary policy needs to play a role in stimulating economic recovery when the economy encounters a serious negative impact. Since the recurrent outbreak of COVID-19 has caused great obstacles to the normal economic exchanges between countries, it has become particularly important to build the domestic market and optimize the industrial allocation at this time. This paper focuses on studying the dynamic impact of China's monetary policy implementation on the industrial structure during the pandemic. Based on the data of the eight major economic zones in Mainland China and the dataset containing many of China's macroeconomic variables, a SV-TVP-FAVAR model is established. The manuscript compares the time-varying effects of monetary policy tools on the industries at different stages before and after the epidemic. The study supported some interesting conclusions. (1) Either the quantitative or price-based monetary policy shocks have significant time-varying impacts on the industries in different economic zones. The impacts of monetary policy on the primary, secondary, and tertiary industries in each economic zone are uneven. (2) The developed Northern, Eastern, and Southern coastal economic zones in Mainland China are more sensitive to the changes in monetary policy. (3) COVID-19 has brought a tremendous negative shock on the economy, which has destroyed the original steady-state of the economic system and added more uncertainty to the regulatory effect of monetary policy. Compared with other periods in China's economic history that severely negatively impacted (the Southeast Asian financial crisis and the global economic crisis), industries in most economic zones under the COVID-19 epidemic have been affected by monetary policy for a longer lag time. Therefore, for the implementation of monetary policy, at the moment of COVID-19 epidemic, we should pay more attention to the dual-pillar role of macro-prudential regulation, further improve the process of China's interest rate reform, enrich the monetary toolbox, and implement differentiated monetary policies in line with the economic zone's position, to optimize the regional industrial structure, and promote long-term economic growth.

## Introduction

The outbreak and epidemic of COVID-19 have significantly impacted economic activities. To alleviate the epidemic's effect, governments worldwide have introduced a series of measures to stimulate economic recovery. Fiscal policy and monetary policy are the most commonly used macroeconomic control methods. Due to the existence of Ricardian Equivalence, monetary policy is more capable of quickly stabilizing the situation when the economy suffers severe external shocks than fiscal policy (1). Moreover, the COVID-19 has the characteristics of recurrent global outbreaks. Each recurrent outbreak of the epidemic have a significant negative impact on the economy, and will greatly deplete consumer confidence (2). Since the founding of the People's Republic of China, monetary policy has developed from nothing to perfection. Especially after the Economic Reform and opening up, monetary policy has gradually grown from an additional fiscal policy to a series of guidelines, policies, and initiatives with clear policy goals that adapt to domestic and foreign financial development. Each fluctuations ironing of China's economy is inseparable from the macro-control of monetary policy, such as inflation control in 1993, deflation caused by the Asian financial crisis in 1997, four interest rate cuts in the global financial crisis in 2008 to stimulate economic recovery, and release of liquidity under the background of the new crown epidemic in 2020, etc. But unlike the previous crises, the COVID-19 pandemic forced the government to take many measures to quarantine and isolate while using macro-control steps to guide economic recovery (3), which have brought great difficulties to regular economic exchanges between countries. In this context, the concept of domestic monetary circulation has been repeatedly mentioned. The top priorities are establishing and improving the domestic market and updating the industrial layout.

In the post-epidemic era, the governments of various countries have taken measures to stimulate the economy internally and strengthen external prevention and control, making the political and trade environment extremely complicated. Moreover, the trend of COVID-19 is changeable and unpredictable. These factors have exacerbated the difficulty of monetary policy implementation, and the industrial structure and layout will also change. To explore the implementation of monetary policy under the background of COVID-19 and its impact mechanism and dynamic evolution principle on the transformation of industrial structure, this paper establishes a time-varying parameter factor-augmented vector autoregressive model with stochastic volatility (SV-TVP-FAVAR) based on the industrial distribution of the eight economic zones in mainland China. Through the establishment of three-dimensional impulse response in the sampling interval and an impulse response function based on time points, taking the impact of the epidemic as a virtual node, the Southeast Asian financial crisis, and the global economic crisis as historical references, this paper analyzes the driving effect of China's monetary policy implementation on industries in different economic zones in detail. It provides experience and respect for China to optimize the industrial structure and implement the feasible path of monetary policy in the post-epidemic era.

**Figure 1:**
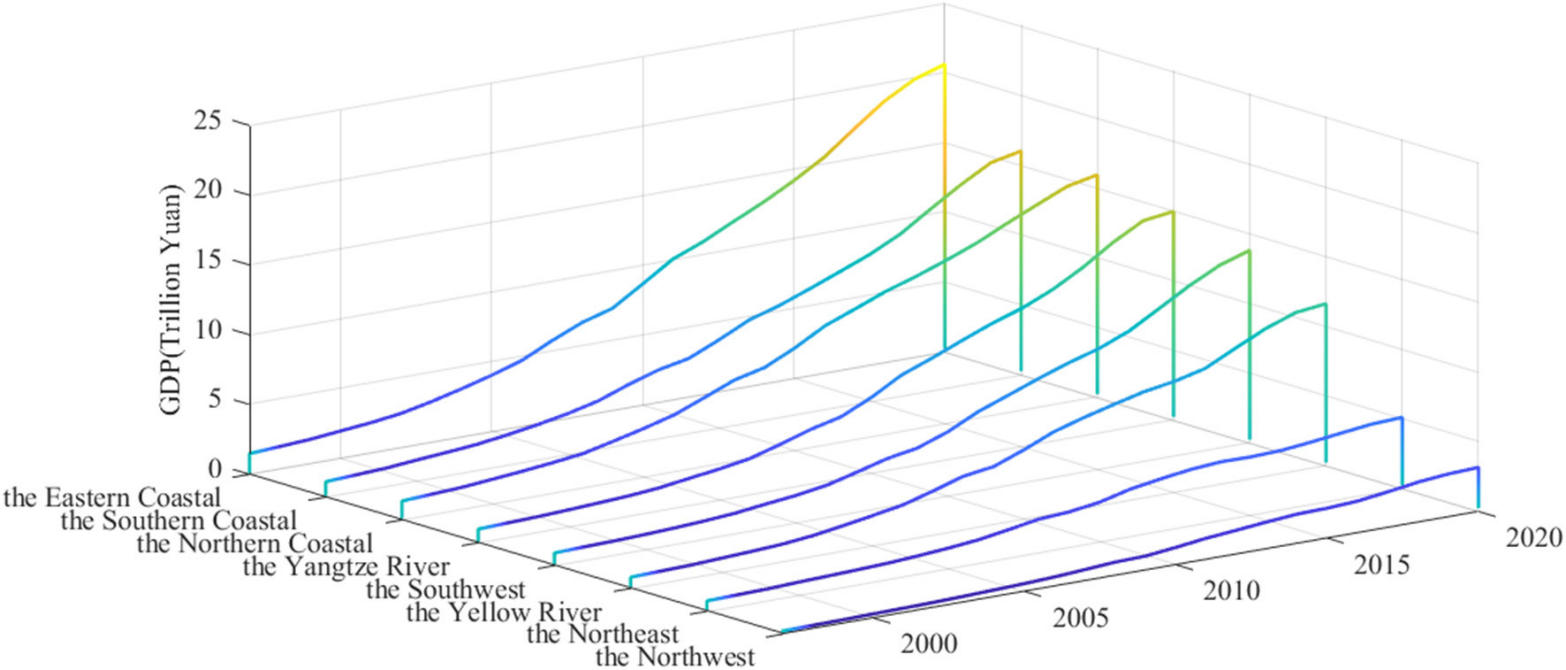
GDP trend of eight major economic zones in mainland China.

On the whole, there is a severe structural imbalance in the distribution of China's industries, and the lack of high-end industries and the overcapacity of low-end industries coexist. Thanks to the effective regulation of the government, the proportion of the tertiary industry have continued to increase in recent years, and the government has made gratifying achievements to a certain extent in restraining backward production capacity and promoting innovation and development. However, there are considerable differences in element endowments, labor composition, natural environment, and industrial layout in different regions due to the vast territory of China. Figure 1 shows the GDP of eight economic zones in mainland China. It can be seen that there are apparent differences in the development level of different economic zones. These differences have hindered coordinated development among regions and further led to an allocation imbalance of financial resources in other economic zones, especially in the context of COVID-19 pandemic; this imbalance will be further exacerbated. Monetary policy can adjust the industrial structure by actively guiding the allocation of element endowments among different industries. Nonetheless, the vast conditional differences in other regions of China objectively lead to the heterogeneous policy effects of a single monetary policy in the different areas. When discussing the industrial structure effect of monetary policies, if external differences such as regional economic development level and financial market organizational structure are not considered, the conclusion will be incomplete. At present, the world economy is in the post-epidemic era. Although governments and central banks have launched a series of fiscal and monetary policies, China faces internal problems such as the disappearance of demographic dividends and the transformation of industrial structure, and external issues such as the impact of trade wars, economic downturn, and pressure of repeated epidemics. Under such circumstances, it is necessary to explore how China's monetary policy guides industrial optimization and how the heterogeneity of policy implementation affects economic growth continuously and healthily.

## Literature Review

Monetary policy is an essential means of national macroeconomic regulation and control. The guiding industrial structure adjustment mechanism is rooted in the uneven distribution of resources among different industries (4). In other words, various industries respond differently to a single monetary policy, and factors of production will be distributed in different sectors under the action of monetary policy (5–9). A complete and reasonable industry chain is one of the critical foundations for realizing technological innovation. Under the background of the epidemic, cross-border communication has suffered a great strike, and the economic growth is weak. It is imperative to explore new endogenous growth points of the economy, optimize, and improve the industrial structure, actively promote sustainable development, and develop high-end industries. Therefore, clarifying the regulatory effect of monetary policy on the industrial structure is an important strategic choice for grasping and deepening China's economic construction. Based on the Ricardo Equivalence theorem (10, 11), monetary policy is more effective than fiscal policy in promoting the industrial structure to optimize and upgrade. The classic literature on the effect of monetary policy on industrial design focuses on verifying its existence and formation mechanism (12, 13). In the process of implementing monetary policy to guide the transformation and upgrading of China's industrial structure, we have to pay attention to one problem: China has a vast territory, and there are differences in objective conditions such as element endowments, industrial layout, and economic development in different regions. Will implementing a unified monetary policy have other policy effects in the various areas? The answer to this question can be traced back to the concept of “Optimal Currency Areas” proposed by Mundell in the 1960s (14). He believed that implementing a unified monetary policy in countries or regions with substantial regional differences is not the optimal solution, and structural effects can interfere with the final policy effect (15–18). Although the criteria for dividing the optimal currency area are almost the same, implementing differentiated monetary policies for areas that do not meet the optimal currency areas can maximize the operating efficiency of the microeconomics and macroeconomics under certain conditions, which has obtained the consensus of most scholars. Chinese scholars start from theoretical and empirical perspectives, analyzing, and comparing the characteristics of different regions in China, believing that China does not meet the optimal currency area hypothesis (19–23). In other words, during the transmission process of a believing monetary policy, due to the friction in the transmission path and the objective conditions of different regions, there will be heterogeneous results in the other areas of China, which will put forward higher requirements for deepening the monetary policy reform. At the same time, it should be noted that the balance of industrial structure between regions does not mean complete homogeneity, and it is equally important to take advantage of different economic zones.

In conclusion, the existing literature has carried out a multi-angle analysis of the monetary policy's effect on the industrial structure and other issues. However, in particular, there are still some deficiencies. First, monetary policy shows apparent asymmetry due to the differences in transmission channels (24–27), so using static or relatively static means to analyze the guiding role of monetary policy on industrial upgrading is not comprehensive. The evasion of the dynamic nature of the monetary policy is one of the reasons why some literature has not reached a unified. Second, monetary policy has a complete target system. When the monetary policy implemented by the central bank acts on the final target through the operation of intermediary indicators, it will affect many economic variables in the financial system. However, the econometric model used in traditional empirical methods can quickly and accurately obtain results when dealing with low-dimensional data. In contrast, it is easy to fall into the “dimension disaster” when facing more variables. The existing literature does not pay enough attention to the large number of variables involved in the process of monetary policy. Third, when monetary policy acts on the industrial structure, China's actual economic situation will inevitably produce structural effects. Therefore, it is not comprehensive to consider monetary policy's guiding role and structural impact in isolation. At present, there is little literature about considering these two aspects' roles into a unified framework. Fourth, the epidemic has caused a large-scale impact on China's economy, politics, and trade. In the complex and changeable post-epidemic era, the dynamic evolution that China's monetary policy impacts industries in different economic zones have not been fully revealed. The implementation of monetary policy requires a more scientific decision-making basis.

Given the shortcomings of the current research, the marginal contribution of this paper is as follows. First, a time-varying parameter factor-augmented vector autoregressive model with stochastic volatility (SV-TVP-FAVAR model) is constructed to replace the classical model, which fully considers the asymmetry of monetary policy in time and structure, using the quarterly data from 1997 Q1 to 2021 Q4 to help observe the dynamic impact of monetary policy on the eight major economic zones in mainland China. Second, the principal component analysis method is used to abstract a large number of economic variables, which contains a lot of information into several unobservable common factors, avoiding the limitation that the model is challenging to deal with high-dimensional variables. Third, while considering the monetary policy to guide the upgrading of the industrial structure, the paper pays attention to the structural effects caused by the transmission mechanism and regional factors and consider them in a unified framework, which provides a richer perspective for monetary policy to guide industrial upgrading and optimization and inter-regional coordinated development. Finally, the epidemic is selected as a virtual event. The Southeast Asian financial and global economic crises are used as comparative events to dynamically clarify the industrial driving effect on China's monetary policy in each economic zone post-COVID-19.

## Model Specification

To avoid the loss of variables in the economic system as much as possible and to deeply analyze the driving effect of monetary policy on industrial structure in different economic contexts, especially during the epidemic, we use the SV-TVP-FAVAR model for empirical research. The SV-TVP-FAVAR model combines the classical VAR model with the factor enhancement idea and uses an innovative random walk method to process the coefficient matrix and the variance-covariance matrix to handle complex time-varying problems. Sims proposed the classical VAR model is widely used in economics because it treats all variables as endogenous variables and avoids the correlation between explanatory variables (28). However, when analyzing macroeconomic issues, especially exploring the dynamic effects of monetary policy on the industries in this paper, the classic VAR model still has many shortcomings. For example, when monetary policy is transmitted to different industries in different regions through channels like interest rates and credit, it will involve much economic information. The traditional VAR model is limited by degrees of freedom and cannot include as many explanatory factors as possible. Hence, it is easy to cause important missing variables (29, 30). Factor enhancement needs to be introduced into the model to eliminate the limitation of degrees of freedom. The classical VAR model is as follows:


(1) $$\begin{array}{l} y_{t}\;=\;\alpha \text{+}\beta_{1}y_{t\;-\;1}\;+\;\ldots \;+\;\beta_{p}y_{t\;-\;p}\;+\;v_{t} \end{array}$$

Where β_1_, β_2_……β_*p*_ are model coefficients, *y*_1_, *y*_2_……*y*_*p*_ are p-order lag term of *y*_*t*_, α is a constant, and *v*_*t*_ ~ *N*(0, Ω _*t*_) is a disturbance term.

It can be seen that the parameter estimation will be limited by the degrees of freedom in the model, and the dimension of $y_{t}^{{\;}^{\prime }}$ is the maximum number of variables that the traditional VAR model can accommodate. This number is generally not higher than 20 and <6 in most cases. Since many indicators are usually needed to consider in studying the dynamic effect of the monetary policy on the industrial structure, many variables involved in the economic process are extracted on this basis, and the high-dimensional matrix is abstracted into several unobservable common factors (29, 30). The Equation used to extract the element is as follows:


(2) $$\begin{array}{l} X_{\text{t}}\;=\;\Lambda^{f}F_{t}\;+\;\Lambda^{y}Y_{t}\;+\;\varepsilon_{t} \end{array}$$

where, *X*_*t*_ is (*N* × 1) dimension database, and is the economic variables of the monetary policy effected on different industries in this paper, *F*_*t*_ and *Y*_*t*_ stand for the part of common factors and proxy variables in the model, respectively, Λ^*f*^ and Λ^*y*^ for (*N* × *K*) and (*N* × *M*)-dimension factor matrix, respectively, *K* and *M* stand for the number of the extracted common factors and the proxy variables of the industries, respectively, and *N* ≫ *K* + *M*., ε_*t*_ ~ *N*(0, Ω_*t*_).

On the other hand, it is necessary to endow the character of time-varying to the model (31, 32) to describe the dynamic impact of quantitative and price-based monetary policies on industrial indicators. Therefore, the following improvements are made on the basis of the classic VAR model:


(3) $$\begin{array}{l} y_{t}\;=\;\alpha \text{+}\beta_{1t}y_{t\;-\;1}\;+\;\ldots \;+\beta_{pt}y_{t\;-\;p}+v_{t} \end{array}$$

where β_*it*_ is a time-varying coefficient matrix (i = 1, 2, ⋯ , *p*), and its residuals have the form of random walks (33). Further, the covariance of disturbance term in Equation (3), then we get:


(4) $$\begin{array}{lll} A_{\text{t}}{\text{Ω}}_{\text{t}}A_{\text{t}}^{{\;}^{\text{′}}}\; & = & \;{\text{Σ}}_{\text{t}}{\text{Σ}}_{\text{t}}^{{\;}^{\text{′}}} \end{array}$$

(5) $$\begin{array}{lll} {\text{Ω}}_{\text{t}}\; & = & \;A_{\text{t}}^{-1}{\text{Σ}}_{\text{t}}{\text{Σ}}_{\text{t}}^{{\;}^{\text{′}}}A_{\text{t}}^{{\;}^{\text{′}}-1} \end{array}$$

where, Σ_*t*_ = *diag*(σ_1, *t*_, ..., σ_*k*+1, *t*_), *A*_*t*_ is a lower triangular matrix with the main diagonal of 1, the form is as follows:


(6) $$\begin{array}{l} A_{t}=\left[\begin{array}{cccc} 1 & 0 & \cdots & 0\\ a_{21,t} & 1 & \ddots & \vdots \\ \vdots & \ddots & \ddots & 0\\ a_{m1,t} & \cdots & a_{m\left(m-1\right),t} & 1 \end{array}\right] \end{array}$$

Rearrange the Equation, where $B_{t}={\left(vec{\left(b_{1t}\right)}^{{\;}^{\prime }},...,vec{\left(b_{pt}\right)}^{{\;}^{\prime }}\right)}^{{\;}^{\prime }}$, $\log\sigma_{t}={\left(\log\sigma_{1t}^{{\;}^{\prime }},...,\;\log\sigma_{mt}^{{\;}^{\prime }}\right)}^{{\;}^{\prime }}$, $\alpha_{t}={\left(a_{j1,t}^{{\;}^{\prime }},...,a_{j\left(j-1\right),t}^{{\;}^{\prime }}\right)}^{{\;}^{\prime }}$, *j* = 1, ..., *m*. Assume the new matrix constituted by *B*_*t*_, α_*t*_ and logσ_*t*_ following the innovation random walk (33):


(7) $$\begin{array}{l} \{\begin{array}{c} B_{t}\;=\;B_{t-1}\;+\;\Gamma_{B}^{t}\;\eta_{B}^{t}\;\\ \alpha_{t}\;=\;\alpha_{t-1}\;+\;\Gamma_{\alpha }^{t}\;\eta_{\alpha }^{t}\;\\ log\sigma_{t}\;=\;log\sigma_{t-1}\;+\;\sigma_{\sigma }^{t}\;\eta_{\sigma }^{t} \end{array} \end{array}$$

where, $\eta_{t}^{\theta }\sim \;N\left(0,Q_{\theta }\right)$ is the innovation variables in the model and are independent of each other, the innovation covariance matrix of *B*_*t*_, α_*t*_ and logσ_*t*_ corresponds to the variance matrix *Q*_θ_ of $\eta_{t}^{\theta }$. When $\Gamma_{t}^{\theta }=0,\forall t=1,...,T$, there is no time-varying part in the estimated parameter, the model returns to the classical constant parameter model. when $\Gamma_{t}^{\theta }=1,\forall t=1,...,T$, it means that the parameters involved in the model are time-varying, and θ_*t*_ ∈ {*B*_*t*_, α_*t*_, logσ_*t*_}.

Further use the lag operator to rearrange the VAR system:


(8) $$\begin{array}{l} y_{t}\;=\;B_{t}\left(L\right)y_{t}\;+\;A_{t}^{-1}\;\underset{t}{\sum }\varepsilon_{t}^{y} \end{array}$$

(9) $$\begin{array}{l} g_{t}\;=\;\Lambda y_{t}\;+\;\Gamma \left(L\right)g_{t}\;+\;W_{t}\varepsilon_{t}^{g} \end{array}$$

where, $g_{t}^{{\;}^{\prime }}\left[x_{t}^{{\;}^{\prime }},z_{t}^{{\;}^{\prime }},C_{t},P_{t}\right]$, $y_{t}^{{\;}^{\prime }}=\left[f_{t}^{{\;}^{\prime }},z_{t}^{{\;}^{\prime }},C_{t},P_{t}\right]$, and *C*_*t*_ is the relatively observed variables of the primary, secondary, and tertiary industries, *P*_*t*_ is instrumental variables of quantitative and price-based monetary policy implement. *W*_*t*_ = *diag*(exp(*h*_1*t*_)/2, ..., exp(*h*_*nt*_)/2, 0_1 × *l*+1_), $W_{t}W_{t}^{{\;}^{\prime }}={\left[H_{t},0_{1\times l+1}^{{\;}^{\prime }}\right]}^{{\;}^{\prime }}$; $B_{t}\left(L\right)\;=\;b_{1t}L+...+b_{pt}L^{p}$; $\left(\varepsilon_{t}^{g},\varepsilon_{t}^{y}\right)$ is the disturbance term, these two are independent and identically distributed and obey the standard normal form; $\Lambda \;=\;\left[\begin{array}{cc} \lambda^{f} & \lambda^{z,C,P}\\ 0_{\left(l+1\right)\times k} & I_{l+1} \end{array}\right]$, λ^*z, C, P*^ = [λ^*z*^, λ^*C*^, λ^*P*^], solve the above Equations (8) and (9) simultaneously:


(10) $$\begin{array}{l} g_{t}\;=\;\tilde{\Gamma }{\left(L\right)}^{-1}\Lambda {\tilde{B}}_{t}{\left(L\right)}^{-1}A_{t}^{-1}\sum_{t}\varepsilon_{t}^{y}+\tilde{\Gamma }{\left(L\right)}^{-1}W_{t}\varepsilon_{t}^{g}\\ \;=\;\Delta_{t}\left(L\right)\varsigma_{t} \end{array}$$

Where ${\tilde{B}}_{t}\left(L\right)\;=\;I\;-\;B_{t}\left(L\right)$, $\tilde{\Gamma }\left(L\right)\;=\;I-\Gamma \left(L\right)$; ς_*t*_ is also an innovation vector that obeys the innovation walk, and is a standard normal distribution. In this way, the SV-TVP-FAVAR model with the idea of factor-augmented and time-varying added to the classical VAR model is constructed.

## Variable Selection

The paper discusses the dynamic role of monetary policy according to the eight economic zones on China's mainland divided by the Development Research Center of the State Council. The variables involved in the model include quantitative and price-based monetary policies economic variables such as the added value of the primary, secondary, and tertiary industries in the eight economic zones. The sampling interval of this paper is selected in consideration of the availability of data and the industrial driving effect of China's actual monetary policy and finally determined the sampling interval from 1997 Q1 to 2021 Q4. The data involved in different economic zones are from the China Economic Net database, and the rest are from the Wind database. All data are quarterly, and some missing data are filled by interpolation. All non-stationary data are converted to stationary form by difference or logarithm.

### Monetary Policy Proxy Variables

According to intermediary variables, monetary policy can be divided into quantitative and price-based. As for quantitative monetary policy, it is necessary to discuss which caliber of the money supply is a proxy variable. Considering that the central bank is the only government agency with the function of currency issuance, through the control of the base currency, the cash M0, M1, and M2 of various calibers can meet the measurability and controllability in the principle of monetary policy intermediary indicators. The problem studied in this paper is the driving degree of monetary policy on the three major industries in each economic zone. However, the scope of cash M0 and M1 is too narrow to reflect society's total demand and purchasing power in real economic life. It is challenging to adjust total supply and demand by M0 or M1. Hence, the quarter-on-quarter added value of M2 is used as a proxy variable for quantitative monetary policy. As for price-based monetary policy, interest rate indicators are highly controllable and measurable. The central bank can easily adjust market interest rates through open market operations or rediscount. Considering that the interbank market can solve the problem of short-term capital shortage and surplus for financial institutions, and has the characteristics of the short loan period, high flexibility, convenient transaction, and interest-rate sensitivity, the interbank market interest rate can more intuitively reflect the supply and demand conditions of the money market. The 7-day interbank rate is selected as the proxy variable of price-based monetary policy, which CHIBOR represents. The proxy variables of monetary policy are all from the China Economic Net database. The selected proxy variables of monetary policy are subjected to the ADF test, and the non-stationary data is logarithmically stable. The statistical description of the data before and after processing is shown in the following Table 1.

**Table 1:** Statistical description of the main variables in the article.

**Var**.	**Sam. per**.	**Mean**	**Max**.	**Min**.	**Std. dev**.	***p*-value**
M2	1996Q4–2021Q4	15.6157	28.9467	8.0333	4.8189	0.9363
D(M2)	1997Q1–2021Q4	−0.0002	0.0547	−0.0257	0.0126	0.0000
CHIBOR	1996Q4–2021Q4	3.5440	12.5567	1.01	2.5405	0.0926
D(CHIBOR)	1997Q1–2021Q4	−0.0013	0.4508	−0.8246	0.2669	0.0136

*Var, variable; Sam. Per, sample period; Max, maximum; Min, minimum; Std. Dev, standard deviation*.

### Variables Related to Each Economic Zone

The eight major economic regions in mainland China are the Northeast, the Northern Coastal, the Eastern Coastal, the Southern Coastal, the Middle Reaches of Yellow River, the Middle Reaches of Yangtze River, the Southwest, and the Northwest Economic Zone. There are considerable differences in objective conditions such as geographical endowment, economic strength, and population size. The three coastal economic zones (the East, the South, and the North) are the most prosperous, with solid economic vitality and complete industrial chains. At the same time, the Northwest Economic Zone has a vast territory and sparsely populated areas, and there is still much room for economic development. We select the primary, secondary, and tertiary industries' added value in each economic zone as the observed variables. The statistical description of the variables is shown in the following Table 2.

**Table 2:** Descriptive statistics of different industries in China's economic zones.

**Economic zone**	**Industry**	**Mean**	**Max**.	**Min**.	**Std. dev**.
The northeast economic zone	Primary industry	3,679.37	7,275.9	1,262.55	2,099.55
	Second industry	111,287.93	17,979.06	3,662.46	5,488.76
	Tertiary industry	11,850.82	26,638.24	2,500.46	8,342.92
The northern coastal economic zone	Primary industry	5,401.89	9,561.69	2,103.62	2,661.56
	Second industry	28,347.07	52,729.84	6,265.38	17,101.77
	Tertiary industry	37,599.50	97,723.68	5,203.00	30,547.38
The eastern coastal economic zone	Primary industry	3,775.89	6,809.52	1,716.29	1,862.58
	Second industry	39,187.29	80,928.85	7,746.53	25,551.87
	Tertiary industry	43,306.04	118,394.3	5,370.04	36,923.23
The southern coastal economic zone	Primary industry	4,091.10	8,638.29	1,683.29	2,231.66
	Second industry	29,779.98	64,834.23	5,007.39	20,758.35
	Tertiary industry	31,397.04	86,724.71	43,84.35	26,398.48
The middle reaches of yellow river economic zone	Primary industry	5,197.01	10,593.08	1,746.51	2,896.77
	Second industry	23,094.72	49,044.49	3,548.92	3,359.36
	Tertiary industry	20,519.79	56,816.22	2,738.77	3,644.89
The middle reaches of yangtze river economic zone	Primary industry	6,639.66	13,798.63	2,643.36	3,627.11
	Second industry	25,695.13	59,915.1	3,488.13	20,447.56
	Tertiary industry	26,389.71	76,080.35	3,473.63	23,889.43
The southwest economic zone	Primary industry	7,062.45	17,054.52	2,429.08	4,591.89
	Second industry	20,496.84	49,170.97	3,569.36	15,678.73
	Tertiary industry	24,399.36	71,881.23	3,067.50	15,139.52
The northwest economic zone	Primary industry	1,738.44	4,002.38	570.98	1,092.63
	Second industry	5,182.87	11,147.24	881.36	3,648.24
	Tertiary industry	6,150.13	16,493.86	885.02	5,198.61

*Max, maximum; Min, minimum; Std. Dev, standard deviation*.

### Macroeconomic Variables Background Database

One of the most important innovations of this paper is the inclusion of as many macroeconomic variables as possible within the analytical framework to avoid the distortion of the industry-driven effect of monetary policy caused by missing essential variables. Concerning classic literature and the availability of data, and considering the final goals, operational indicators, and intermediary indicators in the process of monetary policy implementation, the economic variables of the database in the factor enhancement idea are divided into the following categories. First, macroeconomic regulation and control are ultimately committed to promoting economic growth in actual economic activities. This category includes GDP that measures long-term growth and the GDP of various economic zones in China. Second, the monetary policy implementation process pursued a long-term steady increase in the price circulation category, including GNP deflator, CPI, PPI, and other price indices that measure inflation. Third, in the category of total supply, monetary policy adjustments will affect the total supply, and changes in the supply market will also affect the final policy effect, including labor indicators of each economic zone, etc. Fourth, in the category of financial activity, considering the non-negligible role of financial markets and financial intermediaries in the process of monetary policy transmission, this category covers various calibers of money supply, various stock indexes, various term interest rates, etc. To sum up, 82 economic variables were selected and included in the model information set, and the ADF test was carried out. Difference or logarithmic processing was performed on non-stationary data, X-12 seasonal adjustment was performed on variables with obvious seasonal factors, and data with non-uniform frequencies were interpolated or averaged. After the above processing, all data are quarterly and do not contain first-order unit-roots. The test results are not listed due to space limitations.

## Empirical Results

This section describes the dynamic three-dimensional impulse response among the three industries in different economic zones caused by adjusting various monetary policy tools. A comprehensive analysis of the time dimension and the response dimension discusses the driving effect of monetary policy on the industry. At the same time, to study the changeable impact of monetary policy during the epidemic, the Southeast Asian financial crisis and the global financial crisis are selected as comparisons, research, and compare the similarities and differences in depth of the impact of monetary policy on the industry between the epidemic and the previous economic system which was severely and negatively affected, to provide empirical evidence for the implementation of monetary policy in particular periods.

### Three-Dimensional Impulse Responses of Quantitative Monetary Policy Acting on Different Economic Zones in Mainland China

Taking into account the availability of data and the evolution of China's monetary policy implementation, data from 1997 Q1 to 2021 Q4 is selected as the time interval for the impact of monetary policy on industries in different economic zones and test the dynamic three-dimensional impulse responses of the primary, secondary, and tertiary sectors to quantitative and price-based monetary policies of the eight major economic zones in mainland China, respectively. The X-axis in the figure is the period influenced by the shock, the Y-axis is the time point of the shock, and the Z-axis is the response level of industrial added value in different economic zones to monetary policy, where the X-Z plane constitutes the response dimension of the three-dimensional impulse response. The Y-Z plane includes the time dimension of the three-dimensional impulse response.

**Figure 2:**
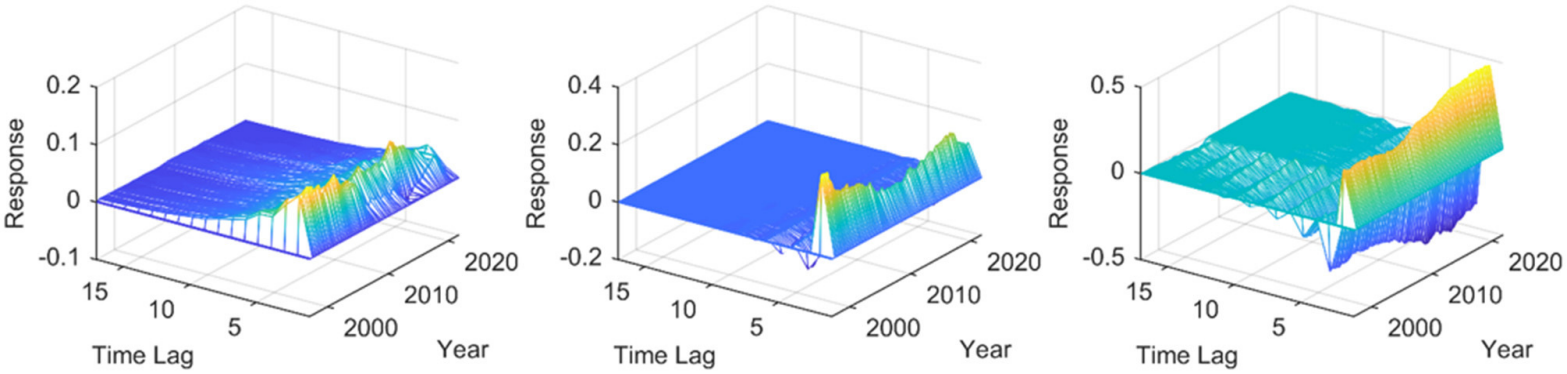
Three-dimensional impulse responses of quantitative monetary policy acting on different economic zones in mainland China. The three sub graphs represent the impulse responses of the primary, secondary, and tertiary industries from left to right, respectively. One period in the figure denotes one season.

First analyze the impact effect of quantitative monetary policy on the primary, secondary, and tertiary industries of mainland China. As shown in Figure 2, expansionary monetary policy has a positive response to all three industries, that is, increasing the money supply can promote the growth of three industries of mainland China in the short term. At the same time, from the perspective of the added value of three industries, the tertiary industry is more than the secondary industry, and more than the primary industry, in other words, the promotion of expansionary monetary policy to three industries is asymmetrical, and there is a trend to promote the transformation into the tertiary industry. China is the largest developing country with a vast territory, and the mainland China has great differences in factor endowments and development among various parts, including the extremely uneven distribution of important resources such as water (34). In order to deeply study the influence of quantitative monetary policy on China's industrial structure, the following is a specific analysis of the dynamic changes facing to the shocks of quantitative monetary policy in different economic zones of mainland China.

Supplementary Figure 1 describes the quantitative monetary policy, researching the dynamic effect caused by one unit of positive shock on the primary, secondary, and tertiary industries in different economic zones in China. Longitudinal comparison finds that primary, secondary, and tertiary sectors in different economic zones do not significantly differ in their response to monetary policy. However, in particular, the responses of industrial added value to quantitative monetary policy do have a considerable difference. As can be seen from Supplementary Figure 1, one unit of shock mainly causes adverse fluctuations in the added value of the primary industry in the Northeast Economic Zone from 1997 Q1 to 2021 Q4. The impact duration is short, and the shock disappears around the 8th period. When the secondary industry in the Northeast Economic Zone is faced with a quantitative monetary policy shock by the same intensity, the impulse response shows more robust heterogeneity. Specifically, one unit of quantitative monetary policy shock on the Northeast Economic Zone's secondary industry-first gives I've downward fluctuations and then upward. The response duration lasts long and returns to the level before the shock at about 12nd. After the shock on the tertiary industry in the Northeast Economic Zone, the maximum response occurs in the second period. The response value is positive, increasing year by year in the time dimension. This shows that both in terms of impact time and impact range, the shocks of quantitative monetary policy cause the most significant fluctuation in the secondary industry in the Northeast Economic Zone, which is consistent with the economic fact that the proportion of the secondary sector in the Northeast industrial base ranks high in the country. The expansionary quantitative monetary policy has a specific positive effect on optimizing the industrial structure in the Northeast Economic Zone. The primary industry in the Northern coastal economic zone has a solid response to the shock of the expansionary quantitative monetary policy. The negative effect was evident before 2000. After entering the twenty-first century, the positive impact gradually prevailed but decreased in 2009 year by year. The response of the secondary industry to the expansionary quantitative monetary policy in the Northern coastal economic zone is slightly weaker than that of the Northeast economic zone. On the whole performs positive fluctuation, which means that the increase of money supply is conducive to raising the secondary industry's added value in the Northern coastal economic zone. The dynamic shocks of the expansionary quantitative monetary policy on the tertiary sector in the Northern coastal economic zone are first negative and then positive, which means that the increase in the money supply will hinder the growth of the tertiary industry in the Northern coastal economic zone in the short term, but stimulate the development of it in the long term. Most of the time, the primary industry in the Eastern coastal economic zone has a negative response to the shocks of quantitative monetary policy. It shows a wave shape that first declines and then rises in the time dimension; that is to say, the effectiveness of driving the primary industry of the expansionary quantitative monetary policy has a weakening trend in the Eastern coastal economic zone. The secondary industry in the Eastern coastal economic zone generally shows an upward fluctuation trend but reaches the maximum positive response value in the third period; there is a time lag in a particular context. The impact of the expansionary quantitative monetary policy on the tertiary industry in the Eastern coastal economic zone is similar to that in the Northern coastal economic zone, which behaves first negative and then positive, indicating that the increase in the money supply of the central bank will be conducive to stimulate the growth of the tertiary industry in the Eastern coast in the long term. The secondary industry in the Southern coastal economic zone response to the expansionary quantitative monetary policy shows a negative effect. The range and trend of response are similar to the primary industry in the Northeast economic zone. The secondary industry in the Southern coastal economic zone is relatively insensitive to the expansionary quantitative monetary policy, which corresponds to the economic fact that the secondary industry in the Southern coastal economic zone accounts for the lowest proportion in the country. The tertiary industry in the Southern coastal economic zone has a more pronounced response to a unit of the positive shock of quantitative monetary policy, showing a slight negative fluctuation at first and then a large positive fluctuation, which supports the conclusion that expansionary monetary policy is beneficial to the tertiary industry in the Southern coastal economic zone in the long term. The response of the primary industry to expansionary quantitative monetary policy in the middle reaches of the Yellow River and the middle reaches of the Yangtze River economic zone is different from the four economic zones mentioned above. The primary industry has a positive added value, which means that the increase of money supply has a driving effect on the Yellow River's middle reaches and the Yangtze River economic zone. Among them, the secondary industry's added value in the middle reaches of the Yellow River economic zone has strong heterogeneity when facing the shock of expansionary quantitative monetary policy, which is characterized by alternating positive and negative responses and a long duration. The tertiary industry in the middle reaches of the Yellow River economic zone showed a response trend of first falling and then rising, reaching the maximum negative response value at the second period and the maximum positive response value at the third period. From the perspective of a time dimension, the negative response value of the tertiary industry to expansionary quantitative monetary policy has an increasing trend in the middle reaches of the Yellow River economic zone after the global economic crisis. The secondary industry has a negative response to expansionary quantitative monetary policy in the middle reaches of the Yangtze River economic zone. The tertiary industry has a negative response first and then a positive response, which the maximum value of the positive response has an increasing trend. The response of the primary industry to the expansionary quantitative monetary policy in the Southwest economic zone shows a trend of rising first and then falling. The maximum value of the positive response increases with time. The secondary industry's added value has strong heterogeneity and a small response range, which is related to the underdevelopment of the secondary industry in the Southwest economic zone. The added value of the tertiary industry-first decreases and then increases when facing a unit of the positive impact of quantitative monetary policy. Supporting quantitative currency has a short-term inhibitory effect on the tertiary industry in Southwest China but has a long-term driving impact. The primary industry in the Northwest economic zone shows a positive response to the expansionary quantitative monetary policy. Yet, the response is minor, related to the weakness of the primary industry in the Northwest economic zone. The secondary industry positively responds to the quantitative monetary policy, indicating that increasing the money supply can promote the secondary industry in the economic area. The tertiary industry-first reacts negatively and positively to the expansionary quantitative monetary policy, which means that the expansionary quantitative monetary policy promotes the tertiary industry's construction in the Northwest economic zone in a relatively long period.

### Three-Dimensional Impulse Responses of Price-Based Monetary Policy Acting on Different Economic Zones in Mainland China

**Figure 3:**
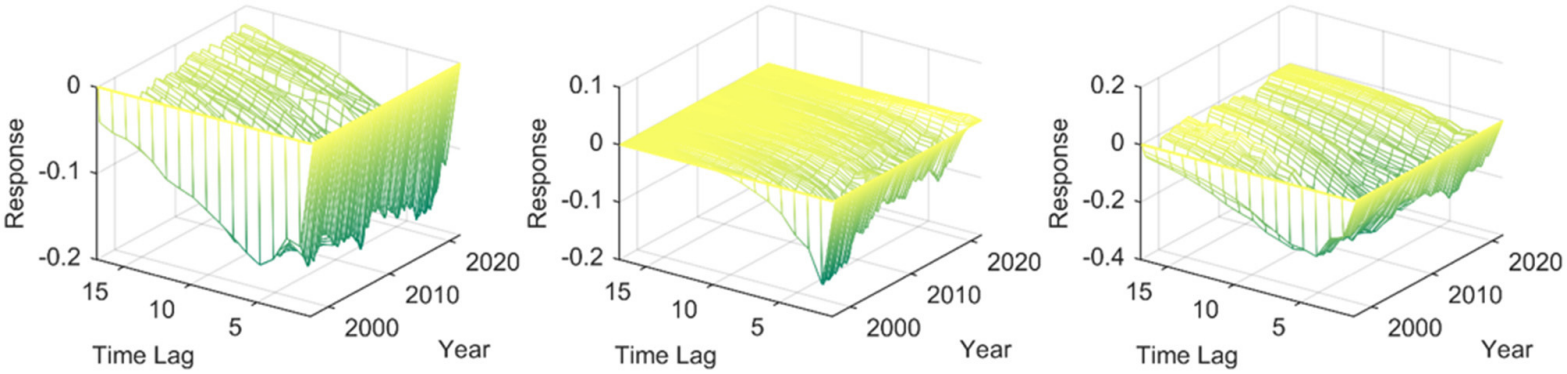
Three-dimensional impulse responses of price-based monetary policy acting on mainland China. The three sub graphs represent the impulse responses of the primary, secondary, and tertiary industries from left to right, respectively. One period in the figure denotes one season.

Further analysis of the impact of price-based monetary policy on the three industries in mainland China. As shown in Figure 3, for the three industries, the contractionary price-based monetary policy causes a negative reaction on the primary industry, the responses range is different, and the tertiary industry has the largest response, which means that the negative impact of increasing the interest rate on the tertiary industry is the most obvious, which is consistent with China's actual economic reality. Supplementary Figure 2 depicts the dynamic effects of a unit of price-based monetary policy on the primary, secondary, and tertiary industries in different economic zones in China. By comparison, it is found that other industries in each economic zone respond to a similar positive impact by the same unit of shock by price-based monetary policy. The growth of each economic zone will be suppressed by the contractionary price-based monetary policy, suggesting that rising interest rates may cause the weakening of industrial development in most economic contexts. In particular, the first, second, and tertiary industries in the Northeast Economic Zone all showed a negative response to the contractionary price-based monetary policy. The primary industry reaches the maximum negative response value in the second period. It returns to the state before the shock at about the 10th period, which means that the contractionary price-based monetary policy will not cause permanent influence to the primary industry in the Northeast economic zone. Within the sampling interval, the secondary industry in the Northeast economic zone has a brief positive response to the contractionary price-based monetary policy in the early stage. Then the response turns negative and increases year by year. The tertiary industry in the Northeast economic zone also shows a negative impulse response to the contractionary price-based monetary policy, indicating that raising interest rates may hinder the growth of the value of the tertiary industry in the Northeast. The Northern coastal economic zone also negatively responds to a unit of the positive shock by price-based monetary policy. The difference is that when the tertiary industry in the Northern coastal economic zone faces a contractionary price-based monetary policy, it takes longer to calm down from the impact. It is related to the relatively developed tertiary industry in the Northern coastal economic zone. The primary industry in the Eastern coastal economic zone generally responds negatively to the contractionary price-based monetary policy, and there is a specific time lag. The effect of price-based monetary policy is longer than that of the Northeast economic zone and the Northern coastal economic zone. The impact of the contractionary price-based monetary policy on the secondary industry in the Eastern coastal economic zone is negative most of the time, and it is increasing year by year in the time dimension. When the tertiary industry in the Eastern coastal areas faces a unit of the positive impact of price-based monetary policy, it produces a short-term positive reaction. It then turns into a negative value at random. If the monetary policy is tightened in recent years, it will negatively impact the tertiary industry in the Eastern coastal economic zone. The Southern coastal economic zone is one of mainland China's most energetic economic zones. The horizontal comparison shows that the added value of the primary, secondary, and tertiary industries in the Southern coastal economic zone is more sensitive to the impact of price-based monetary policy than other economic zones. Specifically, the primary industry in the Southern coastal economic zone has strong heterogeneity in its response to the contractionary price-based monetary policy, which is characterized by frequent switching between positive and negative directions, and the duration of the answer is longer at the beginning of the sampling interval, then it takes 15 periods to recover. The impact of the price-based monetary policy on the primary industry in the Northern coastal economic zone will gradually decrease year by year, and the shock at 2021 Q4 will end around the 8th period. The secondary industry in the Southern coastal economic zone has an obvious negative response to the increase in interest rates, and the negative response value increases in the time dimension. The tertiary industry in this economic zone also negatively responds to the contractionary price-based monetary policy but shows a decreasing trend in the time dimension. Judging from the response mode of one unit of the positive shock of price-based monetary policy in the middle reaches of the Yellow River economic zone, the primary industry of the economic area has a positive response to the impact of the contractionary monetary policy, and the secondary and tertiary industries have a negative response. To a certain extent, it shows that if there is a shock of contractionary price-based monetary policy, it will not be conducive to upgrading the industrial structure in the middle reaches of the Yellow River economic zone. From 1997 Q1 to 2021 Q4, one unit of price-based monetary policy shock also mainly causes negative responses on the primary, secondary, and tertiary industries in the middle reaches of the Yangtze River economic zone, that is, the central bank implementing a contractionary price-based monetary policy mainly causes the negative response of the three industries in the middle reaches of the Yangtze River economic zone. The impact lasts for a long time and calms after about 12 periods. When the Southwest and Northwest economic zones face one unit of the positive shock of price-based monetary policy, the secondary and tertiary industries show obvious negative responses. The negative reaction of the added value of the secondary industry increases in the time dimension. From the response dimension perspective, the tertiary industries in the Southwest and Northwest economic zones are longer affected by the impact than the secondary industry.

### Quantitative Monetary Policy Affects the Industrial Structure of Different Economic Zones in Particular Period

The above three-dimensional dynamic impulse response analyzes the response effects of different industries in the eight major economic zones of mainland China to quantitative and price-based monetary policy tools from a time-varying perspective. It can be seen that quantitative monetary policy causes a more substantial dynamic effect on the industries in each economy zone, and there is significant heterogeneity in the impact on different industries. While price-based monetary policy mainly has a negative shock on various industries in each economic zone, the contractionary monetary policy will directly reduce the money supply and affect the enthusiasm of enterprises for investment, resulting in a negative response to the industrial added value. From the perspective of the time dimension, the impact duration of price-based monetary policy on various industries is longer than that of quantitative monetary policy in most economic contexts.

However, except for research from a time-varying perspective, to measure the driving effect of monetary policy on industries in various economic zones, it should also focus on the changes in industries driven by the monetary policy under particular economic backgrounds. Based on the situation, the next part will start on the epidemic, taking 2020 Q1, the outbreak of COVID-19 as a specific period, and selecting the period of the Southeast Asian financial crisis (1997 Q3). The global economic crisis (2008 Q3), as a representative of the particular period when the economy suffered from severe external shocks, quantifies the changes in industrial added value in different economic zones caused by monetary policy shocks from the actual data level. To make the study of driving effects of quantitative and price-based monetary policy on industries more precise, and provide some empirical evidence for the direction of monetary policy implementation when the economic system is severely negatively affected.

**Figure 4:**
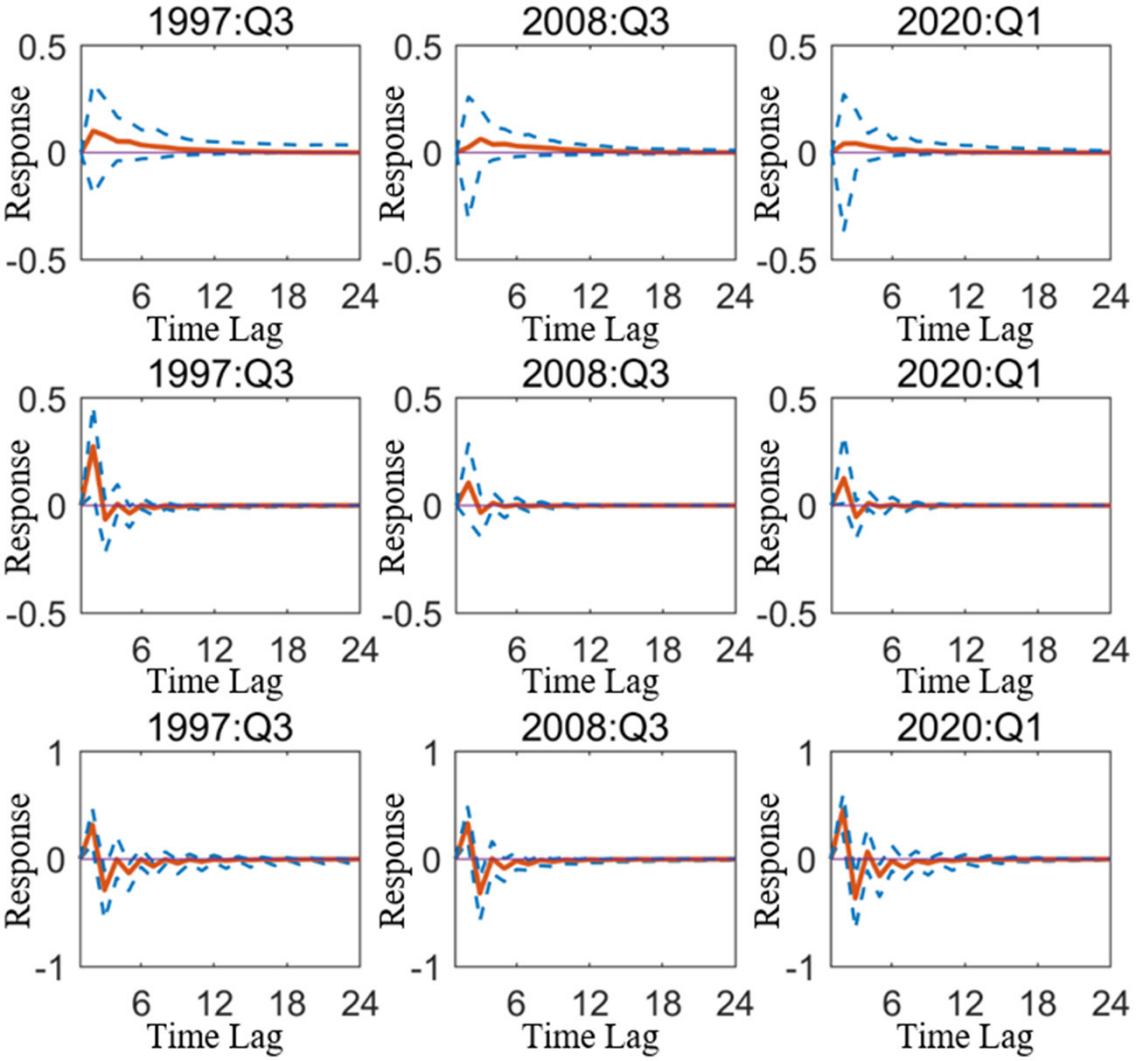
Impulse response of quantitative monetary policy acting on mainland China in particular period. The three sub graphs represent the impulse responses of the primary, secondary, and tertiary industries from top to bottom, respectively. One period in the figure denotes one season.

Figure 4 depicts the impact of quantitative monetary policy on China's primary, secondary, and tertiary industries in the special period, which shows that the primary and secondary industries were more affected by the impact of monetary policy during the Southeast Asian financial crisis, and the tertiary industry was more affected during the COVID-19 epidemic. This means that since the Southeast Asian financial crisis, China's industrial structure has undergone significant change and assumes the incline to the tertiary industry of the industrial structure, resulting in the largest response of the tertiary industry to the impact of one-unit quantitative monetary policy during the epidemic in the three selected special periods.

Supplementary Figure 3A shows the impulse response to one unit of the positive shock of quantity monetary policy during the Southeast Asian financial crisis, the global economic crisis, and the Northeast Economic Zone epidemic. The epidemic period causes the most significant fluctuations of quantitative monetary policy on the primary industry in the Northeast economic zone. The second period reaches the maximum negative response value and disappears at the eighth period. The increase of money supply has an uneven impact on the primary, secondary, and tertiary industries, and the added value of the secondary and tertiary sectors may squeeze out the primary industry. The secondary industry in the Northeast economic zone has the most apparent response to quantitative monetary policy during the Southeast Asian financial crisis and the weakest response during the global economic crisis. The tertiary industry has a significant gap in the three selected special periods, and the Southeast Asian financial crisis and the global economic crisis upward.

The responses of the Northern coastal economic zone to one unit of the positive shock of quantitative monetary policy during the selected three special periods are shown in Supplementary Figure 3B. It can be seen that in the selected three special periods, the primary industry in the Northern coastal economic zone shows a positive response to the expansion of the money supply. It reacted positively to the global financial crisis and reached the maximum positive response value in the second period. Still, from the perspective of the response range, the response value of the secondary industry in the Northern coastal economic zone during COVID-19 is noticeably higher. For the tertiary industry, the shock of expansionary quantitative monetary policy leads to more obvious heterogeneity in its response. The tertiary industry in the Northern coastal economic zone fluctuated negatively during the Southeast Asian financial crisis. During the epidemic, the response of the tertiary industry in the Northern coastal economy is first downward and then upward, and the magnitude is significantly higher than the previous two special periods. By comprehensively comparing the responses of the three industries in the Northern coastal economic zone to one unit of the shock of quantitative monetary policy, it can be found that the response of the industry during the epidemic increased significantly, suggesting that the monetary policy changes during this period have a more noticeable effect on the Northern coastal economic zone.

The results of the response to expansionary quantitative monetary policy tools in the Eastern coastal economic zone in the selected three special periods are shown in Supplementary Figure 3C. It can be seen that in the period of the global economic crisis and the epidemic, the primary industry in the Eastern coastal economic zone has a similar response to the expansionary quantitative monetary policy. From the perspective of the response dimension, we can find that it takes longer for the shock to calm down during the global economic crisis; that is to say, the exact size of the shock of quantitative monetary policy lasts longer during the global economic crisis in the Eastern coastal economic zone. The response of the tertiary industry in the Eastern coastal economic zone to the quantitative monetary policy is first negative and then positive, which means that during the selected three special periods, the expansionary quantitative monetary policy has a short-time negative impact and returns positive in mid-term and long-term on the added value of the tertiary industry in the Eastern coastal economy. The response in the Eastern coastal economic zone during the epidemic is most violent and lasts the longest.

Supplementary Figure 3D shows that for the primary industry in the Southern coastal areas, the response to one unit of the positive shock of quantitative monetary policy during the Southeast Asian financial crisis is greater than that of the same scale during the global economic crisis and the COVID-19. We are observing the response of the secondary industry in the Southern coastal economic zone to the expansionary quantitative monetary policy, indicating that the response in different periods reaches the maximum negative response value in the second period. Overall, the expansionary quantitative monetary policy has a specific inhibitory effect on the development of the secondary industry in the Southern coastal economic zone during the three particular economic periods. The impulse response analysis of the special period supports that the secondary industry in the Southern coastal economic zone is more affected by the impact of the expansionary quantitative monetary policy during the epidemic, and the effect lasts for a longer time. Among them, the response during the epidemic is still the largest.

Observing the response of the primary industry in the economic zone in the middle reaches of the Yellow River to one unit of positive shock of quantitative monetary policy in Supplementary Figure 3E, it can be found that in the three selected special periods, the impact is first positive and then negative, and the response of the epidemic is negatively higher than that of the Southeast Asian financial crisis and the global economic crisis. The trend that the secondary industry in the middle reaches of the Yellow River economic zone response to the expansionary quantitative monetary policy is relatively similar, and turn positive after reaching the maximum negative value in the second period. The response of the tertiary industry to quantitative monetary policy in the middle reaches of the Yellow River economic zone is the period of the epidemic, the Southeast Asian financial crisis, and the global economic crisis in descending order. The impact of the epidemic lasts for a relatively longer period.

The response of the middle reaches of the Yangtze River economic zone to the quantitative monetary policy in the period of the Southeast Asian economic crisis, the global economic crisis, and the epidemic are shown in Supplementary Figure 3F. The comparison shows when the primary industry in the Middle Reaches of the Yangtze River economic zone is shocked by the expansionary quantitative monetary policy, a positive response is generated first and then gradually adjusted to a steady-state The response of one unit of shock on the secondary industry in the middle reaches of the Yangtze River economic zone caused by quantitative monetary policy is the strongest in the Southeast Asian financial crisis, and the corresponding duration of quantitative monetary policy impact lasts for a long time. Similar conclusions also apply to the tertiary industry in the middle reaches of the Yangtze River economic zone.

The results for the response of different industries in the Southwest economic zone to one unit of the positive impact of quantitative monetary policy are shown in Supplementary Figure 3G. It can be seen that the primary industry of the Southwest Economic Zone is affected by the expansionary quantitative monetary policy, first fluctuates upward, and then reaches the peak of positive response in the second period, and then fluctuates downward during the period of the Southeast Asian financial crisis, the global economic crisis, and COVID-19. The expansionary quantitative monetary policy causes the response of the secondary industry in the Southwest economic zone, and the differences are evident among the selected three special periods. During the Southeast Asian financial crisis, the secondary industry in the Southwest economic zone had a positive response followed by a negative response. In the global economic crisis, the response rate is relatively flat, and the response is positive and then negative. At a period of the epidemic, the magnitude of the positive response of the secondary industry in the Southwest economic zone is higher than that of the other two selected economic zones, and the duration is also longer.

Supplementary Figure 3H shows the response to one unit of positive shock on primary, secondary, and tertiary industries in China's Northwest region of quantitative monetary policy during the Southeast Asian financial crisis, the global economic crisis, and the epidemic. It can be seen that the primary industry of the Northwest economic zone has the lightest impact by the expansionary quantitative monetary policy in the three selected special periods, which is consistent with the development status of the Northwest economic zone. The shocks of expansionary quantitative monetary policy on the secondary industry of the Northwest economic zones are first positive and then negative during the selected particular period and are most affected during the epidemic. For the tertiary industry in the Northwest economic zone, in the three selected special periods, firstly fluctuated negatively and then positively fluctuated, which means that during the period of the Southeast Asian financial crisis, the global economic crisis, and the epidemic, the expansionary quantitative monetary policy has an inhibitory effect on the tertiary industry in the Northwest economic zone.

### Price-Based Monetary Policy Affects the Industrial Structure of Different Economic Zones in Particular Period

**Figure 5:**
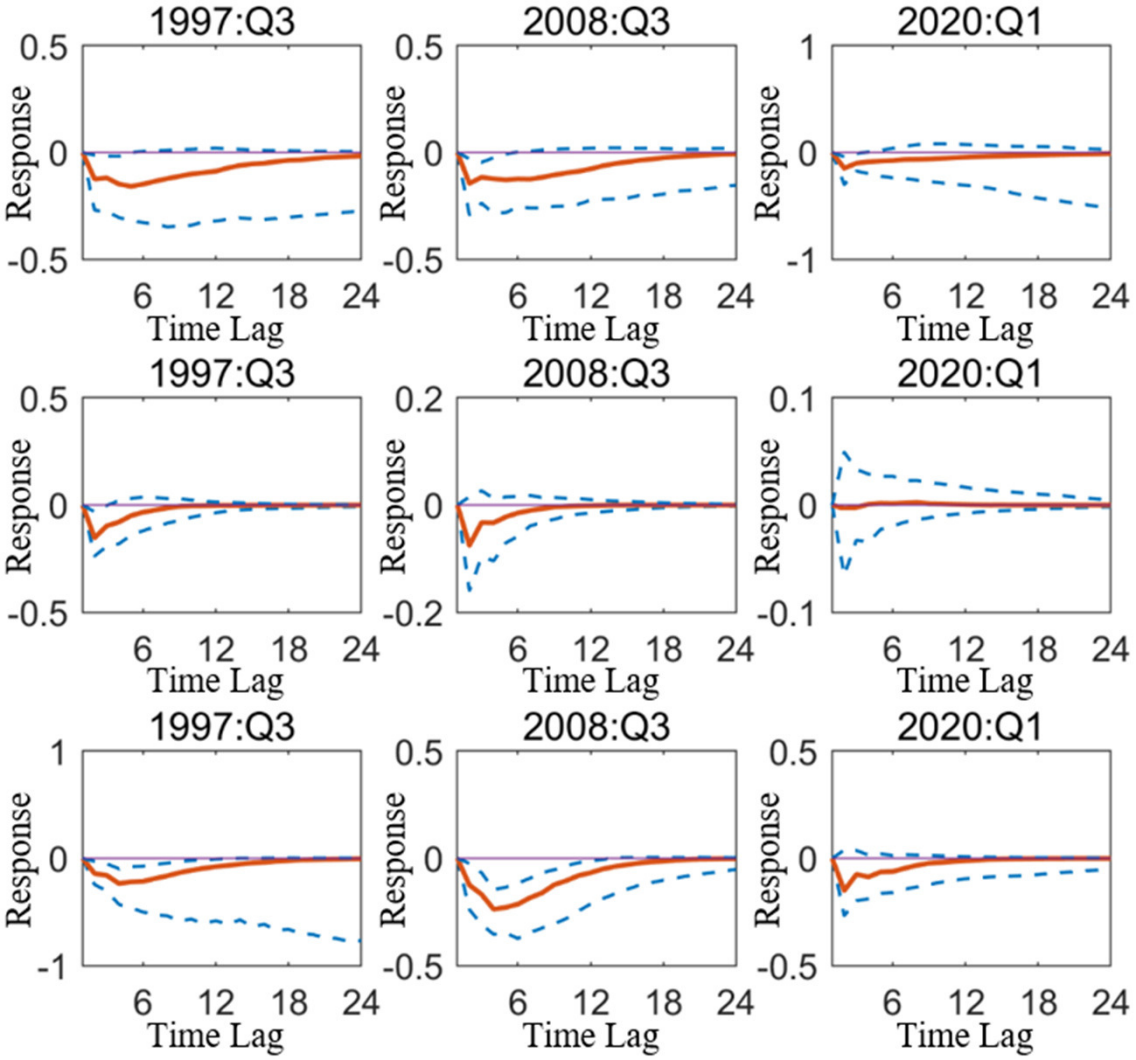
Impulse response of price-based monetary policy acting on mainland China in particular period. The three sub graphs represent the impulse responses of the primary, secondary, and tertiary industries from top to bottom, respectively. One period in the figure denotes one season.

Figure 5 depicts the impact of price-based monetary policy on China's three industries. Overall, contractionary price-based monetary policy will cause negative fluctuations in the three industries during all special periods. For the three industries, the impact of price-based monetary policy is more durable than quantitative monetary policy, which means that the use of interest rate to regulate the national industrial structure is easier to obtain long-term results.

The results that different industries in the Northeast economic zone respond to a unit of the positive shock of contractionary price-based monetary policy are shown in Supplementary Figure 4A. It can be seen that the primary industry in the Northeast economic zone is affected by the contractionary price-based monetary policy, making an adverse reaction during the period of the Southeast Asian financial crisis, the global economic crisis and the epidemic, which means that the increase in interest rates in the selected three special periods will lead a specific inhibition to the development of the primary industry in the Northeast economic zone. The contractionary price-based monetary policy causes the response of the secondary industry in the Northeast economic zone, and the differences are obvious in the three selected special periods. The magnitude of the positive reaction of the secondary industry in the Northeast economic zone is higher than that of the other two selected economic zones, and the response duration was also longer in the epidemic. Horizontal comparison with the response of the tertiary industry in the Northeast economic zone to the shock of the contractionary price-based monetary policy among the Southeast Asian financial crisis, the global economic crisis, and the COVID-19, it can be seen that the tertiary industry in the Northeast economic zone tends to respond negatively during the selected particular period. The response rate during the epidemic is significantly higher.

The response of the Northern coastal economic zone to the impact of price-based monetary policy during the Southeast Asian economic crisis, the global economic crisis, and the epidemic is shown in Supplementary Figure 4B. The comparison shows that when the contractionary price-based monetary policy impacts the primary industry in the Northern coastal economic zone, it produces a negative reaction. Among them, the magnitude and time of the response during the epidemic are more potent than those in the Southeast Asian financial crisis and the global economic crisis, suggesting that during the epidemic, the impact of price-based monetary policy on the primary industry in the Northern coastal economic zone may be more sensitive than that in other two special periods. The response of the secondary industry in the Northern coastal economic zone to a unit of the positive shock of price-based monetary policy is the strongest during the epidemic, and the impact lasts for a long time. Similar conclusions also apply to the tertiary industry in the Northern coastal economic zone.

Observing the response of the primary industry in the Eastern coastal economic zone to one unit of the positive impact of price-based monetary policy in Supplementary Figure 4C, it can be found that in the three selected special periods, the impact is negative. The secondary industry in the Eastern coastal economic zone is most affected by the contractionary monetary policy during the epidemic. The response of the tertiary sector in the Eastern coastal economic zone to price-based monetary policy ranged from large to small in the period of the epidemic, the global economic crisis, and the Southeast Asian financial crisis. The tertiary industry is negatively shocked by the contractionary price-based monetary policy, which negatively affects the industry during the global economic crisis and the COVID-19 epidemic. By contrast, it generates positive volatility in the Southeast Asian financial crisis.

As shown in Supplementary Figure 4D, the three industries in the Southern coastal economic zone face the impact of the contractionary price-based monetary policy, and the trends in the three selected special periods are relatively similar. In-depth analysis shows that for the primary industry in the Southern coastal areas, the response to a unit of the positive shock of price-based monetary policy during the global economic crisis is greater than the same in the Southeast Asian financial and epidemic period. Observing the response of the secondary industry in the Southern coastal economic zone to the contractionary price-based monetary policy, it can be seen that the reaction in different periods reaches the maximum negative response value in the second period. The impulse response analysis of the special period supports that the secondary industry in the Southern coastal economic zone is more affected by the contractionary price-based monetary policy in the period of COVID-19, and the impact lasts for a longer time. For the Southern coastal economic zone, one unit of the shock of price-based monetary policy causes negative fluctuations in its tertiary industry during the three selected special periods.

The responses of the middle reaches of the Yellow River economic zone to the impact of contractionary price-based monetary policy in three selected special periods are shown in Supplementary Figure 4E. It can be seen that the primary industry in the middle reaches of the Yellow River economic zone had a positive impact during the three selected special periods. The secondary industry in the middle reaches of the Yellow River fluctuates positively during the Southeast Asian financial crisis, negatively during the global economic crisis, and the epidemic. Compared with the Southeast Asian financial crisis, there is a specific time lag in emerging the maximum positive value. The response of the tertiary industry to the price-based monetary policy in the middle reaches of the Yellow River Economic Zone is negative first, which means that during the period of the Southeast Asian financial crisis, the global economic crisis, and the COVID-19, the contractionary price-based monetary policy has a short-term negative impact on the added value of the tertiary industry in the Middle Yellow River Economic Zone and the response of the economic zone in the middle reaches of the Yellow River during the epidemic is relatively strong.

The Middle Reaches of the Yangtze River economic zone to a unit of the shock positive of price-based monetary policy in the Southeast Asian financial crisis, the global economic crisis, and the epidemic is shown in Supplementary Figure 4F. It can be seen that in the three selected special periods, the primary industry in the middle reaches of the Yangtze River economic zone has a negative response to the contraction of the money supply. The reaction ranged from large to small in the Southeast Asian financial crisis, the global financial crisis, and the epidemic. The shocks of the same scale of the price-based monetary policy on the secondary industry in the middle reaches of the Yangtze River economic zone are adverse during the three selected particular periods. The contractionary price-based monetary policy shocks lead to more obvious heterogeneity in its response for the tertiary industry. During the Southeast Asian financial crisis, the tertiary industry in the middle reaches of the Yangtze River fluctuated negatively. In the epidemic period, the tertiary industry of the middle reaches of the Yangtze River economic zone first downward and then upward. A comprehensive comparison of the responses of the three industries in the middle goes of the Yangtze River economic zone to one unit of the shock of price-based monetary policy shows that the reaction during the epidemic is more robust than that during the Southeast Asian financial crisis and the global economic crisis, indicating that the effect of the changes of monetary policy on the middle reaches of the Yangtze River economic zone is more prominent.

The Supplementary Figure 4G shows the impulse response to the one-unit positive shock of price-based monetary policy during the Southeast Asian financial crisis, the global economic crisis, and the COVID-19 in the Southwest economic zone. The price-based monetary policy in the selected three special periods causes the negative fluctuations of the Southwest economic zone's primary, secondary, and tertiary industries. For the primary industry, the changes made by the impact of the price-based monetary policy during the global economic crisis are higher than that during the Southeast Asian financial crisis and the epidemic. The secondary industry in the Southwest economic zone has the most apparent response to the price-based monetary policy during the period of COVID-19 and the weakest response during the global economic crisis. The tertiary industry has been affected by the positive shock of price-based monetary policy for the longest time during the Southeast Asian financial crisis.

The responses of different industries in the Northwest economic zone to one unit of the positive shock of price-based monetary policy are shown in Supplementary Figure 4H. It can be seen that the primary industry of the Northwest economic zone is affected by the contractionary price-based monetary policy. During the three selected special periods, the secondary industry in the Northwest economic zone fluctuates downward. Yet, the magnitude of the negative response during the COVID-19 period is higher than that during the global economic crisis. Comparing the response of the tertiary industry in the Northwest economic zone to the shock of the contractionary price-based monetary policy during the period of the Southeast Asian financial crisis, the global economic crisis, and the epidemic, it can be seen that the tertiary industry of the Northwest economic zone tends to be negative during the selected particular period. Among them, the tertiary industry's rate and duration of response in the Northwest economic zone during the epidemic are higher than those during the Southeast Asian financial and global economic crises.

## Conclusion

Starting from the background of COVID-19 pandemic, this paper emphasizes the importance of the government's macro-control, especially monetary policy. And based on the actual economic situation under domestic and international pressure, this paper discusses the necessity and urgency of further optimizing and upgrading the industrial structure. Due to Ricardian Equivalence, monetary policy is a more effective means of regulating the industrial system. The paper establishes the SV-TVP-FAVAR model, taking the eight major economic zones in mainland China as the research object, and profoundly analyzes the dynamic mechanism of monetary policy implementation's impact on different industries in each economic zone. Based on the impulse response function based on time point, the COVID-19 epidemic period is selected as a particular period, and the Southeast Asian financial crisis period and the global economic crisis period are compared and analyzed, focusing on the comparison of time-varying effects on industrial added value between quantitative and price-based monetary policy tools at different stages before and after the epidemic.

The following is based on the analysis of the above empirical results. First, whether a quantitative or price-based monetary policy, the shocks on industries in different economic zones are significantly time-varying. The impact on the primary, secondary, and tertiary sectors in each economic zone is uneven. Among them, the expansion of quantitative monetary policy tends to guide the flow of financial resources into the secondary sector, causing short-term adverse fluctuations, and mid-term and long-term positive fluctuations in the tertiary industry, which means that the positive guiding ability of quantitative monetary policy to the tertiary sector needs to be improved. The tightening of price-based monetary policy will decline industries' added value in all economic zones to varying degrees, and the effect lasts longer. High financing costs will aggravate the financing difficulties of manufacturers in most economic zones and inhibit the desire of enterprises to invest. The impact of the price-based monetary policy affected the secondary and tertiary industries more significantly, which corresponds to the economic fact that the capital of the primary sector is not intensive compared with other sectors, relatively. Second, relatively developed economic zones like Northern coastal, Eastern coastal, and Southern coastal economic zones in mainland China are more sensitive to changes in monetary policy, and the effect of quantitative monetary policy tools is mainly negative in the short term. In relatively developed economic zones, the public tends to think of the increasing money supply as a short-term economic stimulus, so it isn't easy to increase investment and consumption by expanding quantitative monetary policy.

On the contrary, it may lead to price-rising and inflationary pressures, forming a vicious circle. In areas with relatively slow economic growth, such as the Northeast Economic Zone, the Southwest Economic Zone, and the Northwest Economic Zone, the expansion of quantitative monetary policy in these economic zones is conducive to the improvement of infrastructure and related equipment, and it is shown more time positive fluctuations of industrial added value. Third, the influence of monetary policy on the industries of various economic zones under the shock of the epidemic has shown some new characteristics compared with the Southeast Asian financial crisis and the global economic crisis. Specifically, with the spread of COVID-19, the lag time of industries in most economic zones affected by monetary policy will become longer, which means that the epidemic has dramatically disrupted the original steady-state of the financial system, leading to the blockage of monetary policy's transmission channels, and adding more uncertainty to the regulatory effect of monetary policy.

According to the conclusions from the above empirical research, we further propose the following suggestions. The empirical results show that the macroeconomic regulation influence of monetary policy on the industry structure has significant heterogeneity under different economic backgrounds and has apparent structural effects. In general, the regulation effect of price-based monetary policy on the industrial system is much better than that of quantitative monetary policy most of the time. It shows that the process of China's interest rate reform should be further improved, and the economic toolbox should be enriched so that the central bank can be more convenient in formulating relevant macroeconomics monetary policies. Continue to guide the macroeconomics regulation of China's monetary policy from quantitative to price-based, aiming at the different characteristics of economic zones development, implementing differentiated financial procedures matched with the economic zone's position, especially in the epidemic, and more attention should be paid to the two-pillar regulation role of macro-prudence. The regional industrial structure should be further optimized. In particular, the COVID-19 is characterized by repeated outbreaks, and each round of outbreaks is a huge consumption of consumer confidence and GDP. Under such circumstances, it is very difficult to take into account both epidemic prevention policies and economic protection. How to balance the trade-offs is an urgent need for further research in the future.

Although we try to expand the data set affecting the commodity market and stock market as much as possible in this study. There are too many variables involved in the process of monetary policy actions on the industrial structure, and the unavailability of relevant data may limit the scope of the study. With the advancement of statistical methods and computer technology, we look forward to using more advanced methods to study the impact of monetary policy on industrial structure.

## Data Availability Statement

The original contributions presented in the study are included in the article/Supplementary Material, further inquiries can be directed to the corresponding author.

## Author Contributions

SW: conceptualization, methodology, software, formal analysis, data curation, writing—original draft preparation, writing—review, and editing. BZ, SW, HG, and HW: validation. BZ: investigation, resources, supervision, project administration, and funding acquisition. HG: visualization. All authors contributed to the article and approved the submitted version.

## Funding

This research was funded by the Key Project of National Social Science Foundation (Grant No: 20AZD043), the Key Project of Chinese Ministry of Education (Grant No: 17JZD016), and the National Natural Science Foundation of China (Grant No: 11901233).

## Conflict of Interest

The authors declare that the research was conducted in the absence of any commercial or financial relationships that could be construed as a potential conflict of interest.

## Publisher's Note

All claims expressed in this article are solely those of the authors and do not necessarily represent those of their affiliated organizations, or those of the publisher, the editors and the reviewers. Any product that may be evaluated in this article, or claim that may be made by its manufacturer, is not guaranteed or endorsed by the publisher.

## References

[1] Zhang W Zheng J Huang YL. An analysis on anticipated shocks of monetary policy and industrial transmission: based on a multi-sectoral DSGE model. J Fin Res. (2014) 6:33–49.

[2] Han C Li M Haihambo N Babuna P. Mechanisms of recurrent outbreak of COVID-19: a model-based study. Nonlinear Dyn. (2021) 106:1–17. 10.1007/s11071-021-06371-w 33758464 PMC7972336

[3] Wang Q Shi NY Huang JX Yang LQ Cui T Ai J. Cost-effective of public health measure to control covid-19 in china: a microsimulation modeling study. Front Public Health. (2022) 9:726690. 10.3389/fpubh.2021.726690 35059369 PMC8763804

[4] Blinder AS. Credit rationing and effective supply failures. Econ J. (1987) 97:327–52. 10.2307/2232882

[5] Carlino G Defina R. The differential regional effects of monetary policy. J Reg Sci. (1998) 4:572–87. 10.1162/003465398557843

[6] Hayo B Uhlenbrock B. Industry Effects of Monetary Policy in Germany. Munich: University Library Munich (1999) 1:127–58.

[7] Dedola L Lippi F. The monetary transmission mechanism:evidence from the industries of five OECD Countries. Eur Econ Rev. (2005).49:1543–69. 10.1016/j.euroecorev.2003.11.006

[8] Gertler M Karadi P. A model of unconventional monetary policy. J Monet Econ. (2011) 58:17–34. 10.1016/j.jmoneco.2010.10.004

[9] Guo L Liu XH Sun J. Research on Chinese monetary policy and interest rate liberalization—based on equilibrium analysis of economic structure. Econ Res J. (2015) 50:18–31.

[10] Peersam G Smets F. The industry effects of monetary policy in the Euro area. Econ J. (2005) 115:319–42. 10.1111/j.1468-0297.2005.00991.x32046538

[11] Georgopoulos G Hejazi W. Financial structure and the heterogeneous impact of monetary policy across industries. J Econ Bus. (2009) 61:1–33. 10.1016/j.jeconbus.2007.11.003

[12] Larrain M Stumpner S. Capital account liberalization and aggregate productivity: the role of firm capital allocation. J Fin. (2017) 72:506. 10.1111/jofi.12497

[13] Mundell RA. A theory of optimum currency areas. Am Econ Rev. (1961) 4:657–65.

[14] Fleming JM. On exchange rate unification. Econ J. (1971) 81:467–88. 10.2307/2229844

[15] Owyang MT Wall HJ. Structural breaks and regional disparities in the transmission of monetary policy. Federal reserve bank of St. Louis. Work Pap. (2004) 2003–008C. 10.2139/ssrn.927240

[16] Ke DM. The theory of optimum currency areas and its reference to Chinese monetary policy. J Cent Univ Fin Econ. (2001) 1:28–32. 10.3969/j.issn.1000-1549.2001.01.007

[17] Song W Zhong ZS. The existence and origin of regional effects of monetary policy in china—an analysis based on the theory of optimum currency areas. Econ Res J. (2006) 41:13.

[18] Jiao JP Sun TQ Liu XY. On the regional difference in the effectiveness of monetary policy. J Fin Res. (2006) 3:1–15. 10.1088/1475-7516/2006/03/002

[19] Zhou ZL Zhao JS. A review of the theoretical research on regional differentiation of monetary policy—based on the perspective of optimal currency area theory. Wuhan Fin Mon. (2008) 2:33–5.

[20] Zhang H Wang Z. The structural effect of the transmission variables of my country's monetary policy. Econ Perspect. (2013) 4:58–63.

[21] Dai JP Jin YJ. Asymmetric effects of monetary policy on different industry. J World Econ. (2006) 29:46–55+96. 10.1088/1126-6708/2006/07/004

[22] Cao YQ. Asymmetric effects of monetary policy on industries in China. J Quant Tech Econ. (2010) 27:18–30+42.

[23] Wang JB Guo XQ Cai JB. The output overshooting, the consumptive restraint and the inflationary inertia in the context of expansionary monetary policies. Manag World. (2011) 3:7–21.

[24] Liu DP. Analysis on the structural effects of China's monetary policy from dynamic perspective—an empirical analysis based on the TVP-SV-FAVAR model. Stud Int Fin. (2018) 3:25–34.

[25] Sims CA. Comparison of interwar and postwar business cycles: monetarism reconsidered. Am Econ Rev. (1980) 70:250–7. 10.3386/w0430

[26] Sims CA. Drift and breaks in monetary policy. (1980) 7:1–14. Available online at: http://sims.princeton.edu/yftp/Sydney/DriftBreak.pdf

[27] Sims CA Zha T. Macroeconomic Switching. (2002) 2:1–23. Available online at: http://citeseerx.ist.psu.edu/viewdoc/download?doi=10.1.1.199.3593&rep=rep1&type=pdf

[28] Stock JH Watson MW. Implications of dynamic factor models for VAR analysis. Work Pap. (2005) 11467. 10.3386/w11467

[29] Bernanke BS Boivin J. Monetary policy in a data-rich environment *J Monet Econ*. (2003) 50:525–46. 10.1016/S0304-3932(03)00024-2

[30] Nakajima J. Time-Varying parameter VAR model with stochastic volatility: an overview of methodology and empirical applications. Monet Econ Stud. (2011) 29:107–42. Available online at: https://www.imes.boj.or.jp/research/papers/english/me29-6.pdf

[31] Primiceri GE. Time varying structural vector autoregressions and monetary policy. Rev Econ Stud. (2005) 73:821–52. 10.1111/j.1467-937X.2005.00353.x

[32] Negro MD GE Primiceri. Time varying structural vector autoregressions and monetary policy: a corrigendum. Rev Econ Stud. (2015) 82:1342–5. 10.1093/restud/rdv024

[33] Koop G Leon RG Strachan RW. On the evolution of the monetary policy transmission mechanism. J Econ Dyn Control. (2009) 33:997–1017. 10.1016/j.jedc.2008.11.003

[34] Bian DH Yang XH Wu FF Babuna P Luo YK Wang B. A three-stage hybrid model investigating regional evaluation, pattern analysis and obstruction factor analysis for water resource spatial equilibrium in China. J Cleaner Prod. (2022) 331. 10.1016/j.jclepro.2021.129940

