# Supplementary material for: Research on Monetary Policy Implementation and Industrial Structure Transformation Under COVID-19—Evidence From Eight Economic Zones in Mainland China
Source: Front Public Health. 2022 May 20;10:865699. doi: 10.3389/fpubh.2022.865699 (PMC9163316; doi:10.3389/fpubh.2022.865699)
Supplement: Supplementary file 1 [file Image_1.PDF]

Supplementary Material

1 Supplementary Figures

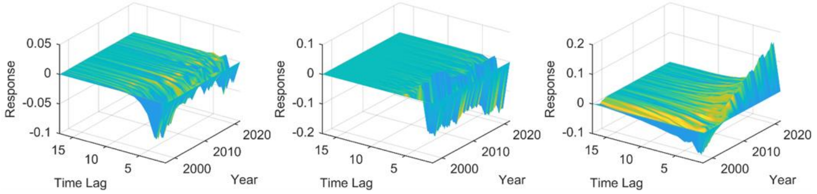

(a) The Northeast Economic Zone

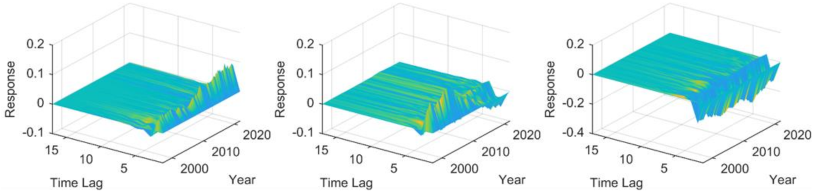

(b) The Northern Coastal Economic Zone

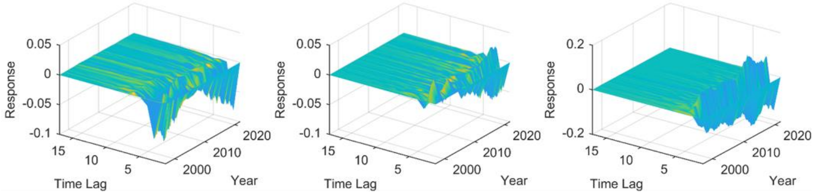

(c) The Eastern Coastal Economic Zone

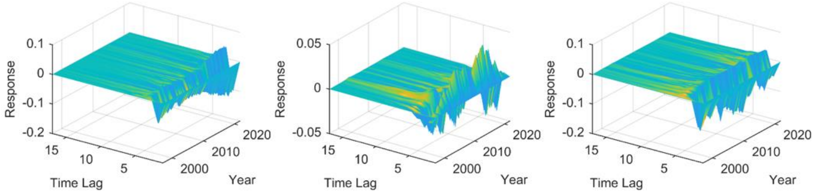

(d) The Southern Coastal Economic Zone

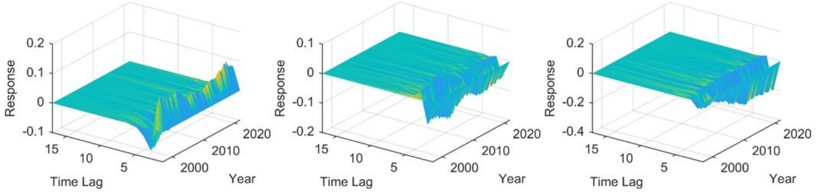

(e) The Middle Reaches of Yellow River Economic Zone

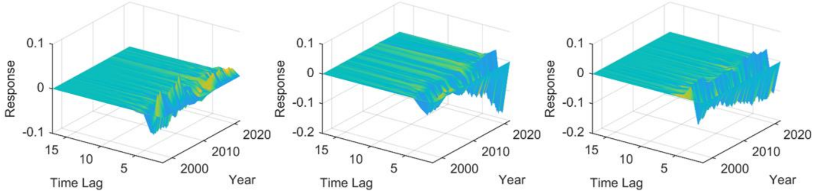

(f) The Middle Reaches of Yangtze River Economic Zone

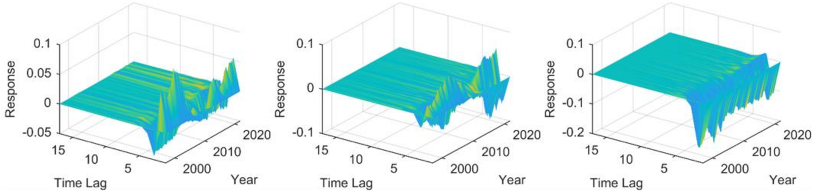

(g) The Southwest Economic Zone

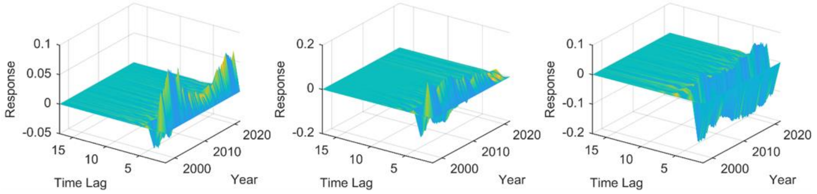

(h) The Northwest Economic Zone

Supplementary Figure 1. Three-dimensional impulse responses of quantitative monetary policy acting on different economic zones in mainland China. Note: The three subgraphs represent the impulse responses of the primary, secondary, and tertiary industries from left to right, respectively. One period in the figure denotes one season.

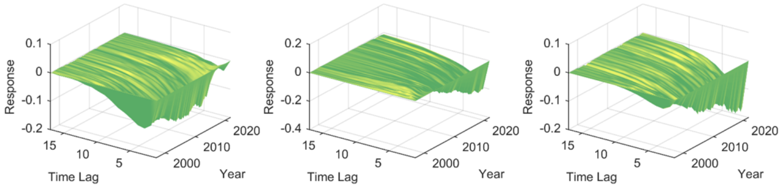

(a) The Northeast Economic Zone

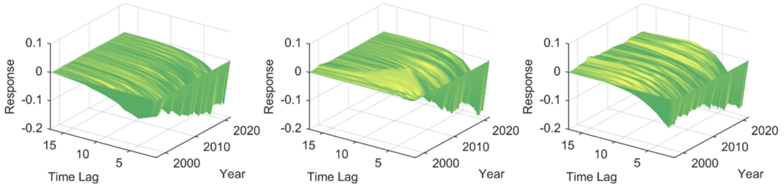

(b) The Northern Coastal Economic Zone

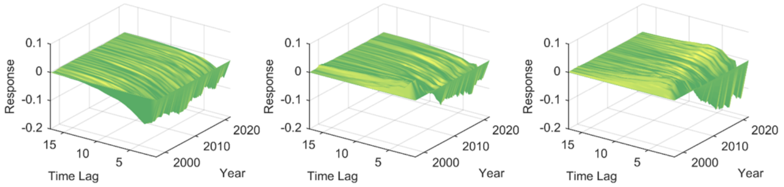

(c) The Eastern Coastal Economic Zone

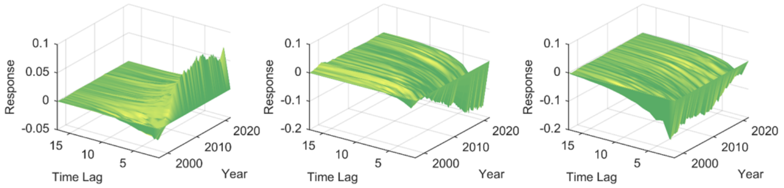

(d) The Southern Coastal Economic Zone

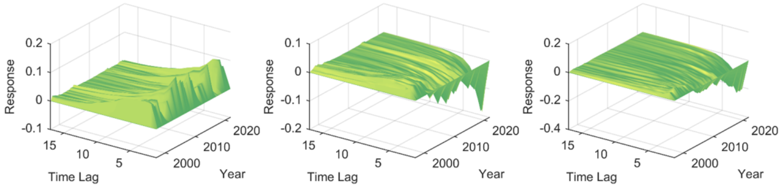

(e) The Middle Reaches of Yellow River Economic Zone

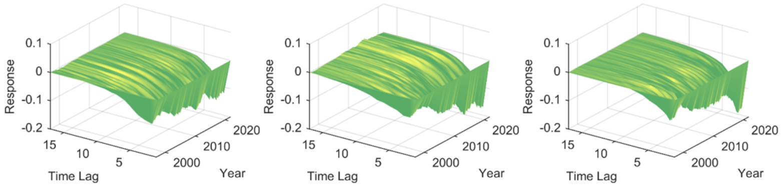

(f) The Middle Reaches of Yangtze River Economic Zone

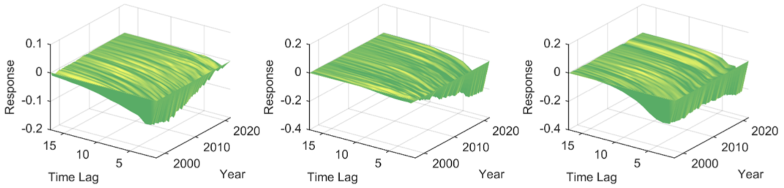

(g) The Southwest Economic Zone

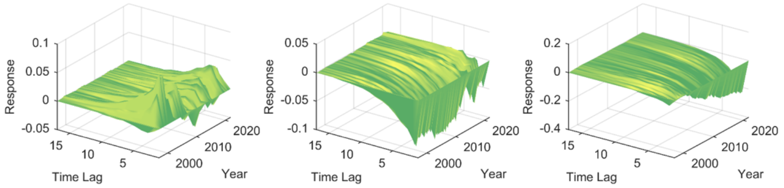

(h) The Northwest Economic Zone

Supplementary Figure 2. Three-dimensional impulse responses of price-based monetary policy acting on different economic zones in mainland China. Note: The three subgraphs represent the impulse responses of the primary, secondary, and tertiary industries from left to right, respectively. One period in the figure denotes one season.

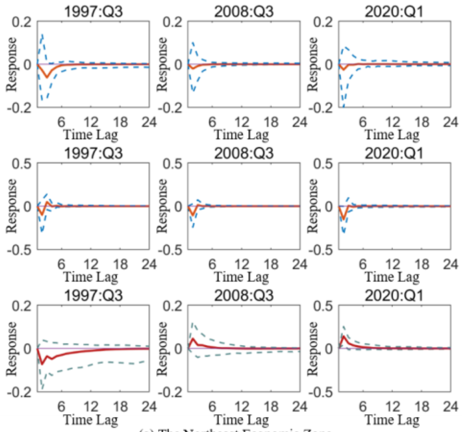

(a) The Northeast Economic Zone

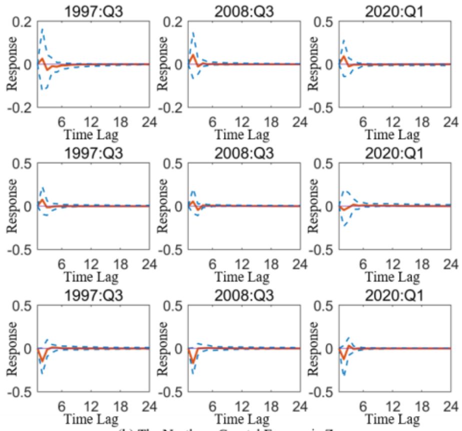

(b) The Northern Coastal Economic Zone

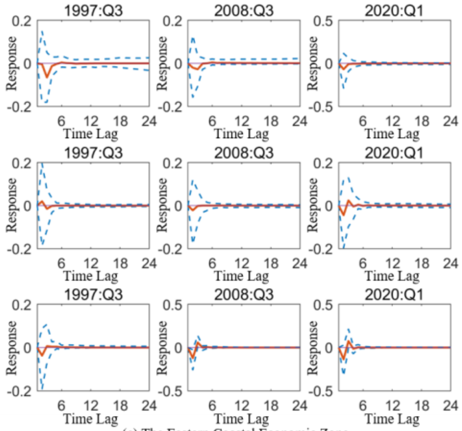

(c) The Eastern Coastal Economic Zone

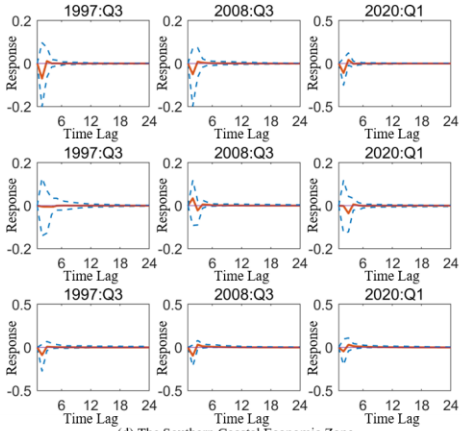

(d) The Southern Coastal Economic Zone

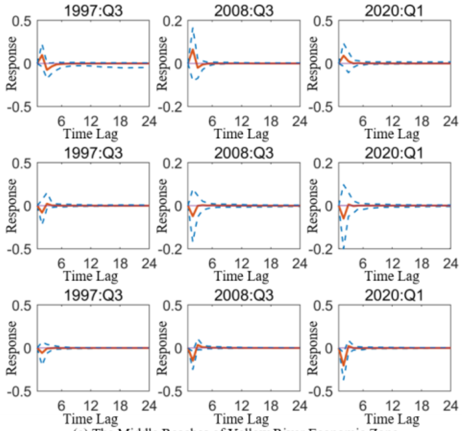

(e) The Middle Reaches of Yellow River Economic Zone

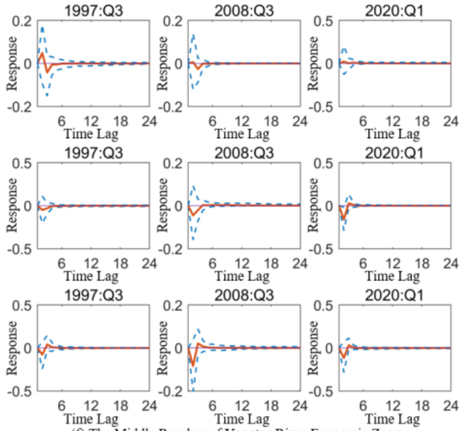

(f) The Middle Reaches of Yangtze River Economic Zone

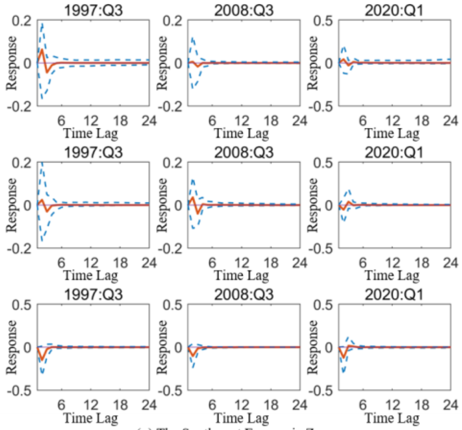

(g) The Southwest Economic Zone

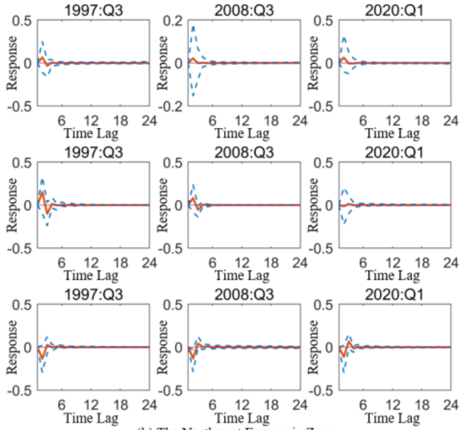

(h) The Northwest Economic Zone

Supplementary Figure 3. Impulse response of quantitative monetary policy acting on different economic zones in mainland China in particular period. Note: The three subgraphs represent the impulse responses of the primary, secondary and tertiary industries from top to bottom, respectively. One period in the figure denotes one season.

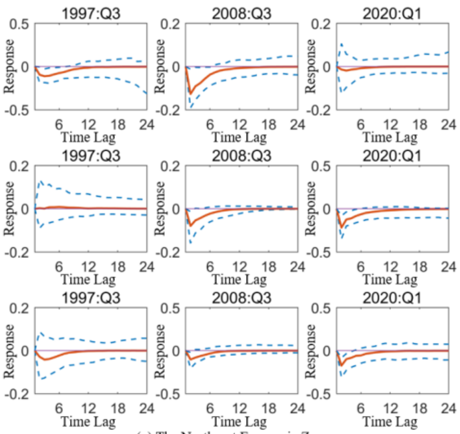

(a) The Northeast Economic Zone

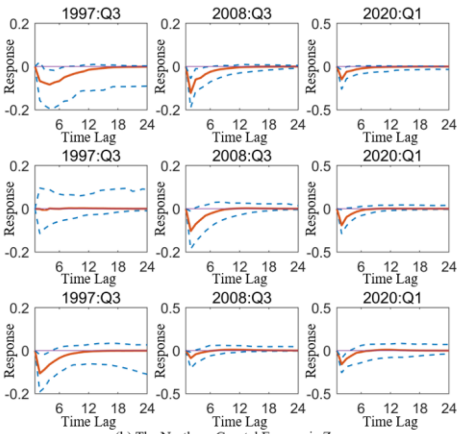

(b) The Northern Coastal Economic Zone

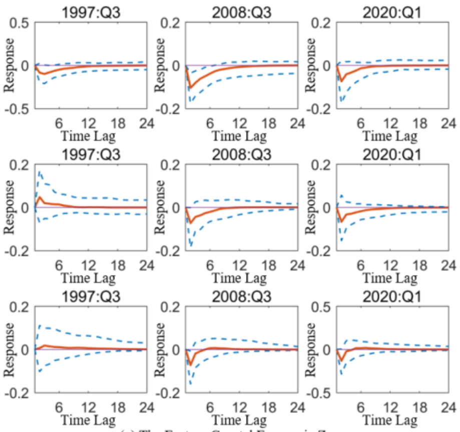

(c) The Eastern Coastal Economic Zone

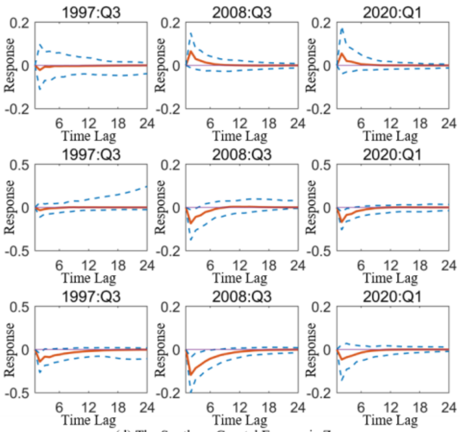

(d) The Southern Coastal Economic Zone

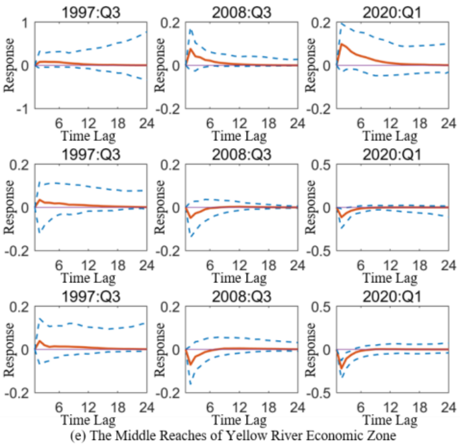

(e) The Middle Reaches of Yellow River Economic Zone

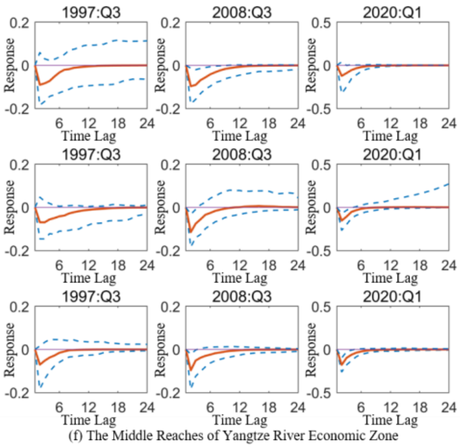

(f) The Middle Reaches of Yangtze River Economic Zone

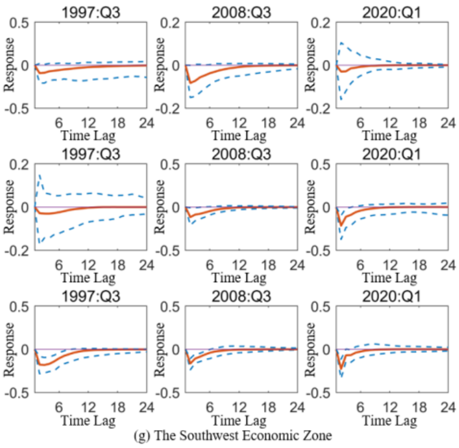

(g) The Southwest Economic Zone

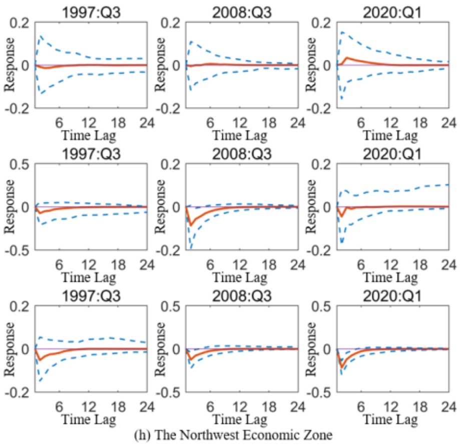

(h) The Northwest Economic Zone

Supplementary Figure 4. Impulse response of price-based monetary policy acting on different economic zones in mainland China in particular period. Note: The three subgraphs represent the impulse responses of the primary, secondary and tertiary industries from top to bottom, respectively. One period in the figure denotes one season.
